# Low cost bio-derived carbon-sprinkled manganese dioxide as an efficient sulfur host for lithium–sulfur batteries†

**DOI:** 10.1039/c8ra03793k

**Published:** 2018-07-04

**Authors:** Aswathy Raghunandanan, Ulaganathan Mani, Ragupathy Pitchai

**Affiliations:** Flow Battery Section, Electrochemical Power Sources Division, CSIR-Central Electrochemical Research Institute Karaikudi-630 003 Tamil Nadu India ragupathyp@cecri.res.in; Academy of Scientific and Innovative Research New Delhi India

## Abstract

Realization of the lithium-sulfur battery system is of major concern because a theoretical cell capacity of 1675 mA h g^−1^ can be obtained at an average voltage of 2.1 V. The primary issues that hinder the practical applications of this system include its poor utilization of sulfur, limited cycle life and retarded rate performance. In the present study, hemp-derived carbon (C-hemp) is made into a composite with room temperature-synthesized MnO_2_, which acts as a host for sulfur in the lithium-sulfur battery system. The composite material is characterized physico-chemically and electrochemically using various techniques. This composite exhibits better electrochemical performance as a sulfur carrier compared to pristine carbon. An initial specific capacity of 926 mA h g^−1^ is obtained at 0.1 C for C-hemp/MnO_2_-sulfur, which surpasses that of the C-hemp-sulfur sample. C-hemp provides a conductive matrix as well as porous sites for the accommodation of sulfur, while MnO_2_ exhibits the ability to absorb polysulfide chemically. Thus, this composite is established as a potential cathode for lithium-sulfur batteries.

## Abstract 2

MnO_2_-biomass (hemp) derived carbon composite is used as an effective cathode in Li–S cell. MnO_2_ acted as polysulfide scuffolding in the composite enhancing Li–S cell performance. New carbon source (hemp-fibre) was utilised successfully in Li–S.

## Graphical abstract

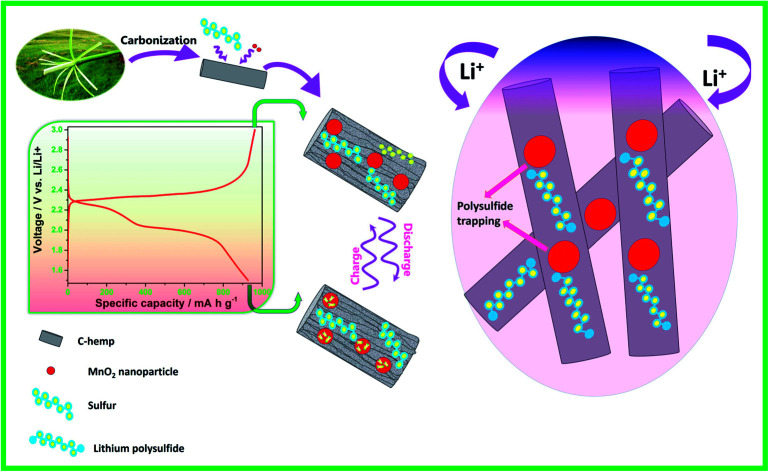

## Introduction

In the 21st century, the critical need for high performance and long-life energy storage systems is highly dependent upon the generation of safe, cost effective and high energy density rechargeable batteries, which are required for the development of electric vehicles, renewable energy plants and smart grids.^1,2^ However, traditional lithium ion batteries fail to meet the requirements because they generate low energy density, utilize raw materials and pose an environmental hazard. This has caused the emergence of other types of batteries such as lithium-air, lithium-sulfur, zinc-air, sodium-ion, and aluminium ion batteries. The conducive characteristics of sulfur such as low cost, non-toxicity, resource abundance make it a promising candidate for next generation energy storage systems.^3,4^ Also, it possess a high theoretical specific capacity of 1675 mA h g^−1^.^5,6^ Furthermore, it delivers a high energy density (2500 W h kg^−1^) when coupled with lithium (Li–S cell), which is much higher than that of other available lithium-ion batteries.^7–9^ However, the commercialization of these batteries is hindered by critical problems. Primarily, the intrinsic insulating nature of sulfur and lithium sulfides lead to a low volumetric energy density, and the large volume change of sulfur and aggregation of the charge discharge products upon cycling lead to a decrease in the electrode stability and internal redox shuttle causing capacity fading, low specific capacity and low coulombic efficiency.^10–12^

Thus, to address the abovementioned hurdles, research is ongoing in the development and modification of sulfur cathodes, lithium anodes,^13^ electrolytes^14^ and separators.^15,16^ The poor conductivity of sulfur can be overcome by impregnating sulfur into conductive matrices of carbonaceous materials such as porous carbon,^17–21^ carbon spheres,^22,23^ carbon fibers,^24–26^ carbon nanotubes,^27–30^ and graphene.^31–36^ Additionally, its electronic conductivity can be effectively enhanced by combination with carbon. The high conductivity of carbon reduces the polarization of the cathode and its high surface area traps the polysulfide intermediates by physical interaction.^37^ Nevertheless, the non-polar nature of carbon has limited ability in suppressing lithium polysulfide dissolution. Accordingly, high performance can be achieved by not only improving the conductivity and reducing polysulfide shuttling, but also the rectification of electrode pulverization.^38^

Another advantageous strategy for superior performance is exploiting the chemical interaction of polar materials with lithium polysulfides.^39–44^ Various polar materials have been utilized for this purpose including TiO,^45^ TiO_2_,^46,47^ Ti_4_O_7_,^48^ MnO_2_,^49–57^ SiO_2_,^58^ Al_2_O_3_,^43,59^ La_2_O_3_,^60^ MgO^61^ and Mg_0.6_Ni_0.4_O.^62^ Additionally, combining carbon materials with manganese oxide not only improves their conductivity but also produces absorption agents for polysulfides. Herein, we report a cathode structure based on δ-MnO_2_-decorated C-hemp as the host material for Li–S rechargeable cells, which shows an appreciable cycling performance and rate capability. The porous nature of C-hemp provides a conductive matrix and ensures the confinement of sulfur in the pores. Additionally, the polar nature of the metal oxide enables the absorption of polysulfide intermediates. The composite matrix also stabilizes the capacity to a small degree.

## Experimental

### Materials

Sublimed sulfur (≥99.5%), bis(trifluoromethane)sulfonimide lithium salt (LiTFSI), anhydrous lithium nitrate, dioxolane (DOL, 99%) 1,2-dimethoxyethane (anhydrous DME, 99.5%), and analytical grade chemicals such as KMnO_4_ were procured from Sigma Aldrich. Polyvinylidene fluoride (PVDF), *N*-methyl pyrrolidinone (NMP) and Super P were purchased from Alfa Aesar. l-glycine was procured from Fischer Scientific. All chemicals were used as received without further purification.

### Synthesis

#### Hemp-derived carbon

This was obtained from the thermal carbonization of hemp fibers (*Cannabis sativa*) followed by activation with alkali. The fibers were washed with water, cleaned and dried at 60 °C for 12 h. Then, the dried fibers were soaked in 10% KOH solution overnight followed by carbonization at 220 °C for 3 h. The obtained product was ground well in an agate mortar and heated in a tubular furnace at 750 °C for 30 min under a nitrogen atmosphere. The product was washed with 0.2 M HCl followed by several washes with distilled water until it reached neutral pH and then dried. The purified carbon material is henceforth referred to as C-hemp.

#### MnO_2_ nanoparticles

MnO_2_ nanoparticles was prepared using a method reported elsewhere.^63^ In the present study, an aqueous solution of a 1 : 1 molar ratio of KMnO_4_ and amino acid was sonicated for 30 min and the brown precipitate formed was allowed to settle. This precipitate was centrifuged and washed with excess water followed by ethanol. Then, it was dried at 80 °C overnight.

#### C-hemp/MnO_2_–S composite

**Scheme 1:**
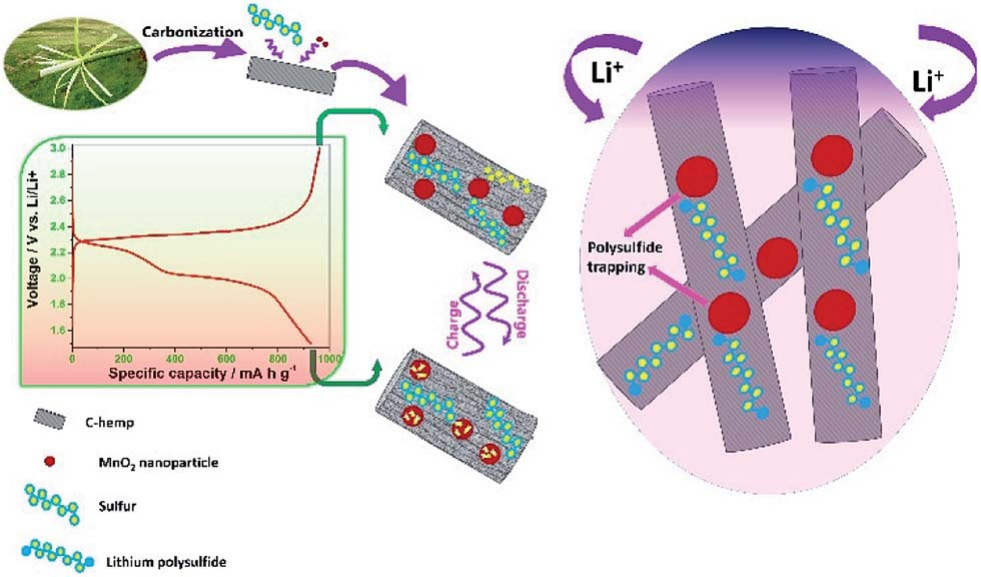
Schematic representation of the C-hemp/MnO_2_–S composite employed as a cathode material for Li–S batteries.

C-hemp and MnO_2_ in a 1 : 2 ratio were dispersed in distilled water by ultrasonication for 1 h and continuously stirred overnight. The nanocomposite was collected by centrifugation, washed repeatedly with water and dried at 70 °C in an oven. Afterwards, a given amount of C-hemp/MnO_2_ and sulfur were mixed well by grinding and heated at 150 °C under an N_2_ atmosphere for 6 h to ensure the uniform impregnation and distribution of sulfur. A schematic representation of the C-hemp/MnO_2_–S composite employed as a cathode material for Li–S batteries is presented in Scheme 1.

### Physico-chemical characterization

The crystal structure and phase purity of the synthesized materials were assessed *via* powder X-ray diffraction (XRD) on a Philips XRD ‘X’ PERT PRO diffractometer with a Cu Kα (*λ* = 1.5418 Å) source. Thermal analysis was carried out in the temperature range from ambient to 700 °C under a nitrogen atmosphere at a heating rate of 10 °Cmin^−1^ using a TA Instruments Model SDT Q600 thermogravimetric analyzer. The chemical state of the surface and the elemental composition of the sample materials were determined *via* X –ray photoelectron spectroscopy (XPS) using a Thermo Scientific MULTILAB 2000 with a Twin Anode Mg/Al (300/400W) X-Ray source and 110 mm radius hemispherical analyser with 7 channeltrons as the detector. The morphological characteristics of the samples were analysed *via* FE-SEM (Carl Zeiss, SUPRA 55VPFEI, Germany). A Denver CE10 (0111) microbalance with 10 μg sensitivity was used for weighing materials and electrodes. Deionized water was used for all experiments.

### Electrochemical characterization

The C-hemp/MnO_2_–S composite was mixed with super P conductive carbon and PVDF in a ratio 7 : 2 : 1 using NMP as the solvent. The slurry was coated on aluminium foil and dried at 50 °C. The electrodes were cut into circular discs with a diameter 15.4 mm and had an average sulfur loading of 3 mg cm^−2^. 2032 coin cells were fabricated in an argon-filled glove box (MBraun, Germany) with the C-hemp/MnO_2_–S composite as the cathode and Li metal as the anode. The electrolyte was composed of 1 M LiTFSI and 50 mM anhydrous LiNO_3_ in a 1 : 1 mixture of DME and DOL. All electrochemical experiments were conducted at room temperature. Cyclic voltammograms were recorded on a Solartron, USA at a scan rate of 0.1 mV s^−1^ between 3 and 1.5 V. Galvanostatic charge–discharge profiles at different current densities were obtained using a computer-controlled battery test system (Arbin, USA) between the voltage range of 1.5 and 3 V. Electrochemical impedance spectroscopy measurements were performed before and after cycling using a Biologic Instrument, France and the resistive parameters were calculated using the Zfit software.

## Results and discussion

**Fig. 1:**
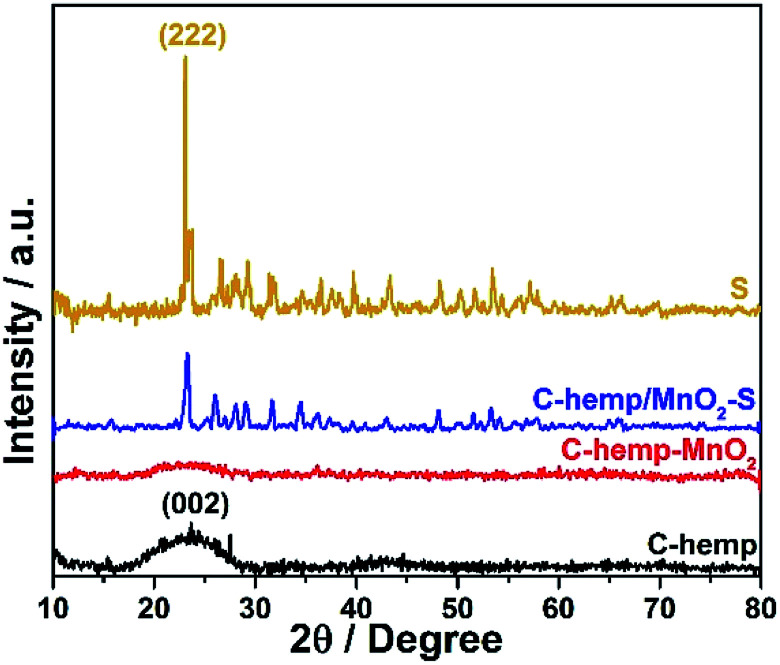
XRD patterns of C-hemp, C-hemp-MnO_2_ and C-hemp/MnO_2_–S.

The structural characteristics of the composite formed were investigated using the XRD technique. The XRD pattern of the sulfur impregnated C-hemp is shown in Fig. 1. The broad peak at around the 2*θ* value of 26° can be assigned to the (002) crystallographic plane of graphitic carbon. The sulfurized sample exhibits well-defined sulfur peaks corresponding to orthorhombic sulfur (JCPDS 00-008-0247), which indicates the presence of sulfur on the surface of C-hemp.

**Fig. 2:**
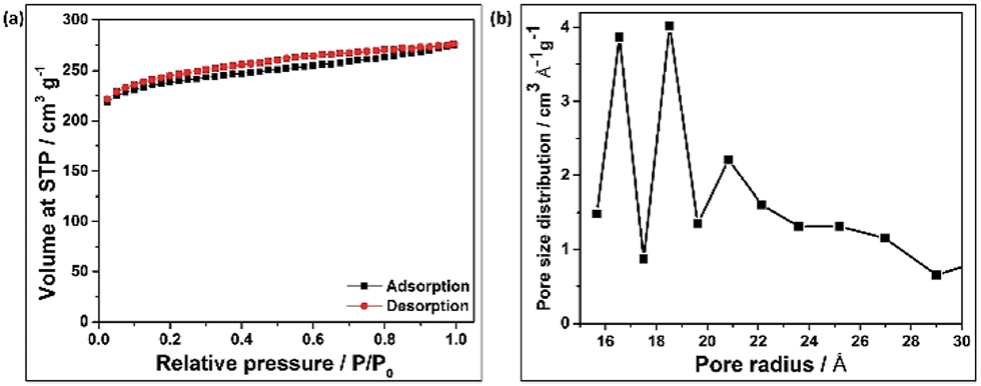
(a) Adsorption/desorption curves of C-hemp. (b) Pore size distribution of C-hemp.

The surface area of C-hemp after activation with alkali was calculated to be 746 m^2^ g^−1^, while that without activation was only 34 m^2^ g^−1^. This enhancement in surface area influences the electrochemical behaviour of the material. Fig. 2a shows the nitrogen adsorption–desorption isotherm, which suggests the type IV nature of the carbon material with mesoporous structures. The mean pore radius of C-hemp is approximately 16 Å, as evident from Fig. 2b.

**Fig. 3:**
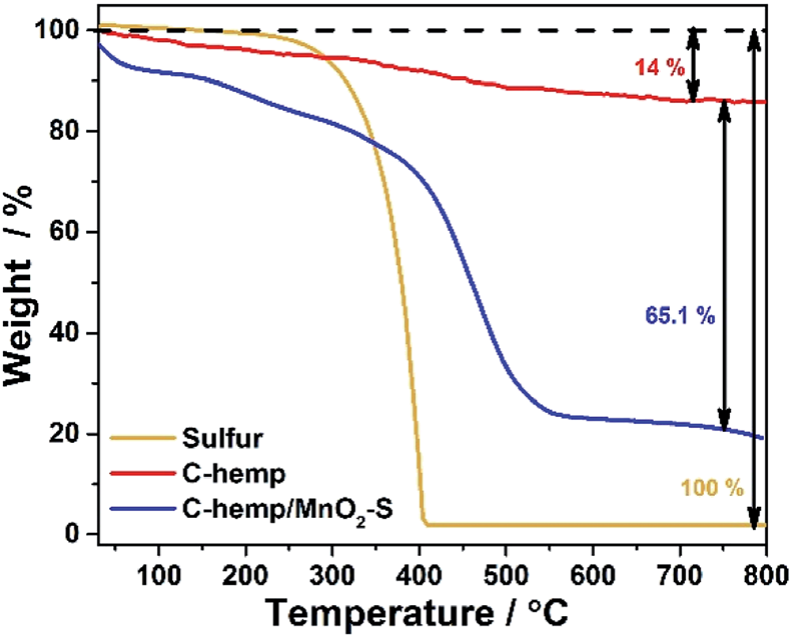
TGA of C-hemp, C-hemp/MnO_2_–S and S.

To confirm the sulfur content, the sample materials were subjected to thermogravimetric analysis and the results are shown in Fig. 3. Considering that MnO_2_ is not likely to feature large weight changes below 700 °C; here, the sulfur loading in C-hemp-/MnO_2_–S is estimated to be ∼65% by TGA. Thus, the porous nature of C-hemp enables the accommodation of the maximum amount of sulfur.

**Fig. 4:**
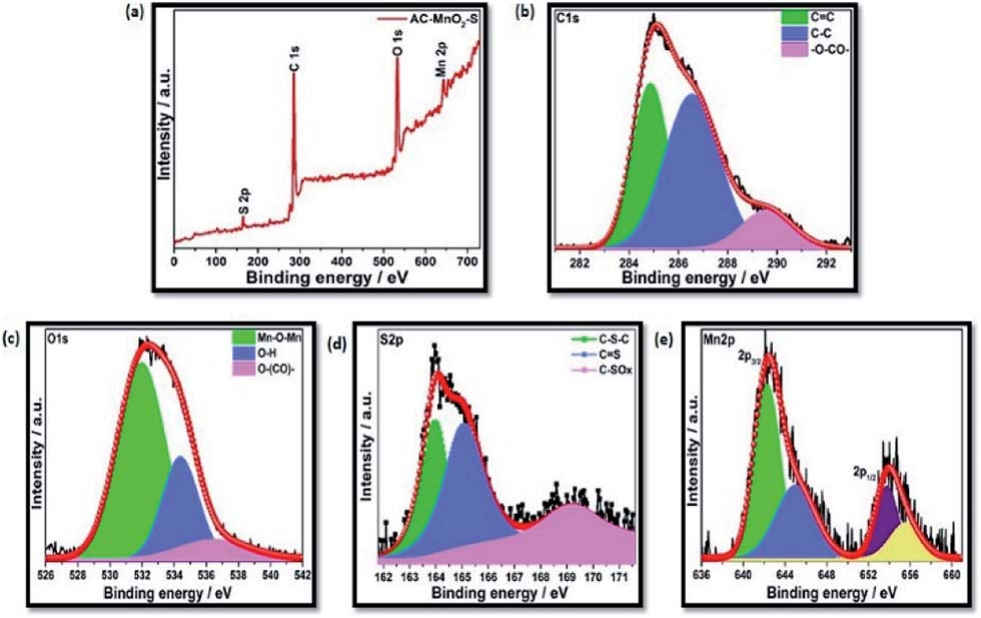
(a) XPS survey spectrum of C-hemp/MnO_2_–S. High-resolution spectra of (b) C1s (c) O1s (d) S2p and (e) Mn2p.

To study the chemical composition and surface properties of the materials, XPS measurements were carried out and the results are displayed in Fig. 4. The survey spectrum given in Fig. 4a evidences the existence of manganese Mn2p and sulfur S2p in addition to carbon C1s and oxygen O1s, which indicate the successful incorporation of manganese and sulfur in the carbon matrix. The peaks centered at approximately 286.0 eV and 533.0 eV in all the survey spectra correspond to C1s and O1s, respectively. For the composite material, C1s is deconvoluted into three peaks at 284.8, 286.4 and 289.5 eV corresponding to C=C, C–C and C–S, respectively, as shown in Fig. 4b.^64–66^ The O1s spectrum shown in Fig. 4c is deconvoluted into three peaks at 531.9 (C=O), 534.3 (C–O) and 536.3 (chemisorbed oxygen or water).^67^Fig. 4d shows that the deconvoluted XPS S2p spectrum is fitted into three peaks positioned at 163.9 (C–S–C), 165.1 (C=S) and 169.1 eV (C–SO_*x*_).^64,68^ In the Mn 2p region shown in Fig. 4e, the 2p3/2 and 2p1/2 doublet is observed at 642.7 and 654.2 eV is consistent with previous reports.^69,70^

**Fig. 5:**
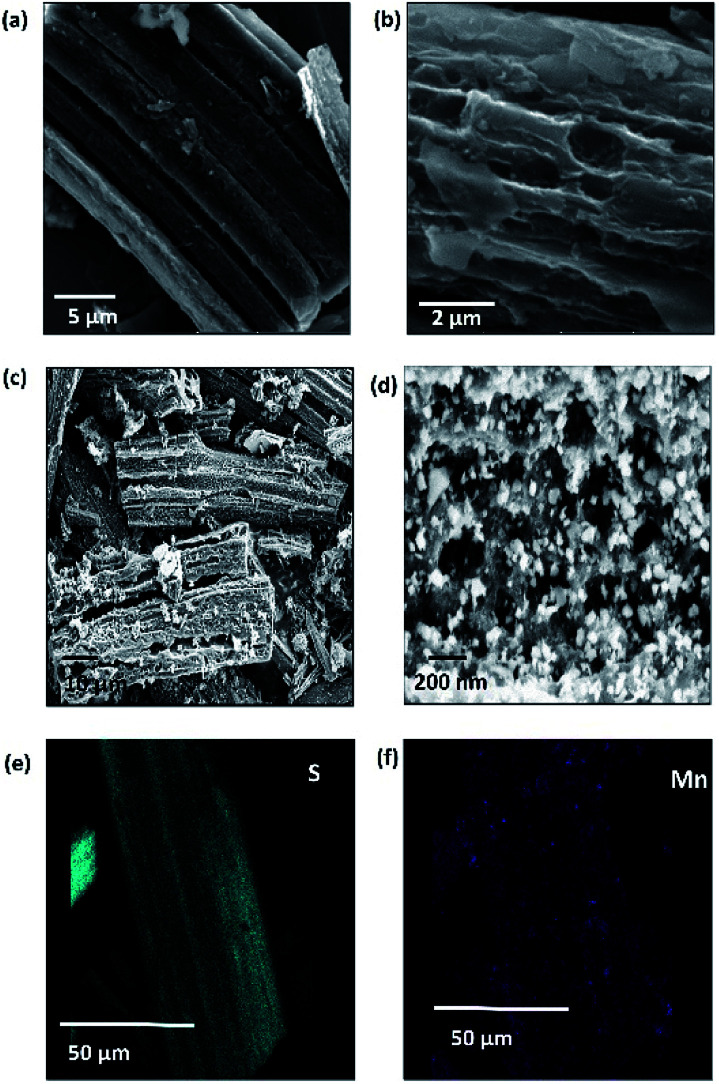
FESEM images of (a) and (b) C-hemp and (c) and (d) C-hemp/MnO_2_–S. Elemental mapping of (e) s and (f) manganese.

The morphology of C-hemp and C-hemp/MnO_2_–S is shown in the FE-SEM images in Fig. 5a–d. C-hemp exhibits a bundle-like morphology with a porous nature, which is visible in Fig. 5a and b. The composite of C-hemp/MnO_2_–S has a similar structure with sulfur coated and impregnated onto the porous C-hemp structure. Granular particles of MnO_2_ nanoparticles are also found all over the carbon substrate. Fig. 5e and f show the energy dispersive spectroscopy mapping profiles of C-hemp/MnO_2_–S. These images show that S and MnO_2_ are uniformly distributed over the C-hemp matrix. This uniform distribution of MnO_2_ is beneficial for the effective trapping of polysulfides.

**Fig. 6:**
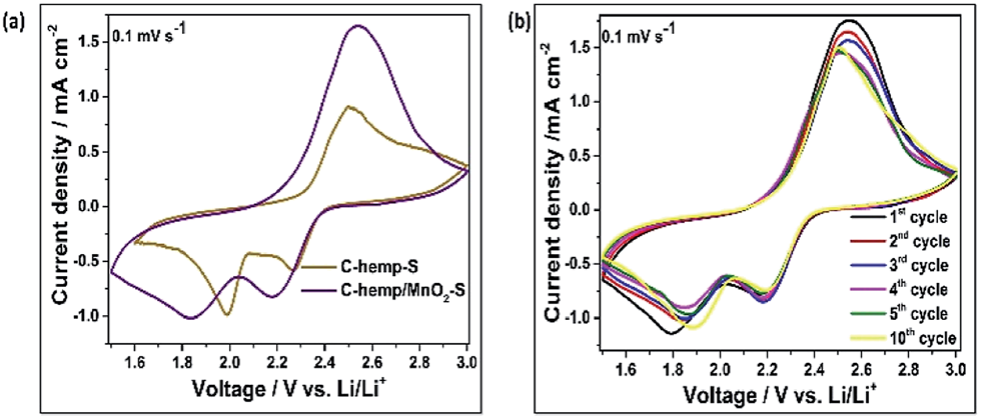
(a) Cyclic voltammograms of C-hemp-S and C-hemp/MnO_2_–S at a scan rate of 0.1 mV s^−1^. (b) Cyclic voltammograms of C-hemp/MnO_2_–S for the first 5 cycles.

The electrochemical performance of the material was assessed using cyclic voltammetry. The electrochemical evaluation of C-hemp-S was evaluated with the same sulfur content for comparison. Fig. 6a shows the comparative cyclic voltammograms of the C-hemp-S and C-hemp/MnO_2_–S cells. The C-hemp-S cathode exhibits typical cathode behaviour with well-defined cathodic and anodic peaks. During the cathodic sweep, two reduction peaks were obtained at 2.3 V and 2.0 V, which suggest the multi-step reduction of sulfur. The first reduction peak at 2.3 V corresponds to the reduction of cycloocta sulfur (S_8_) to higher order polysulfides (Li_2_S_*n*_, 4 ≤ *n* < 8) and that at 2.0 V indicates the decomposition of the long chain polysulfides to shorter polysulfides (Li_2_S_2_ or Li_2_S). During the anodic scan, a single intense oxidation peak was observed at 2.41 V due to the slow kinetics of the oxidation of lithium sulfide to high order lithium polysulfides. In the case of the cathode with MnO_2_ additive, two cathodic peaks and one anodic peak appeared, which were shifted slightly to lower and higher potentials, respectively. This implies that MnO_2_ does not electrochemically participate in the charge–discharge process of the cell. After three consecutive cycles, the anodic peak was stabilized, which indicates the good durability of the material as a cathode (Fig. 6b).

**Fig. 7:**
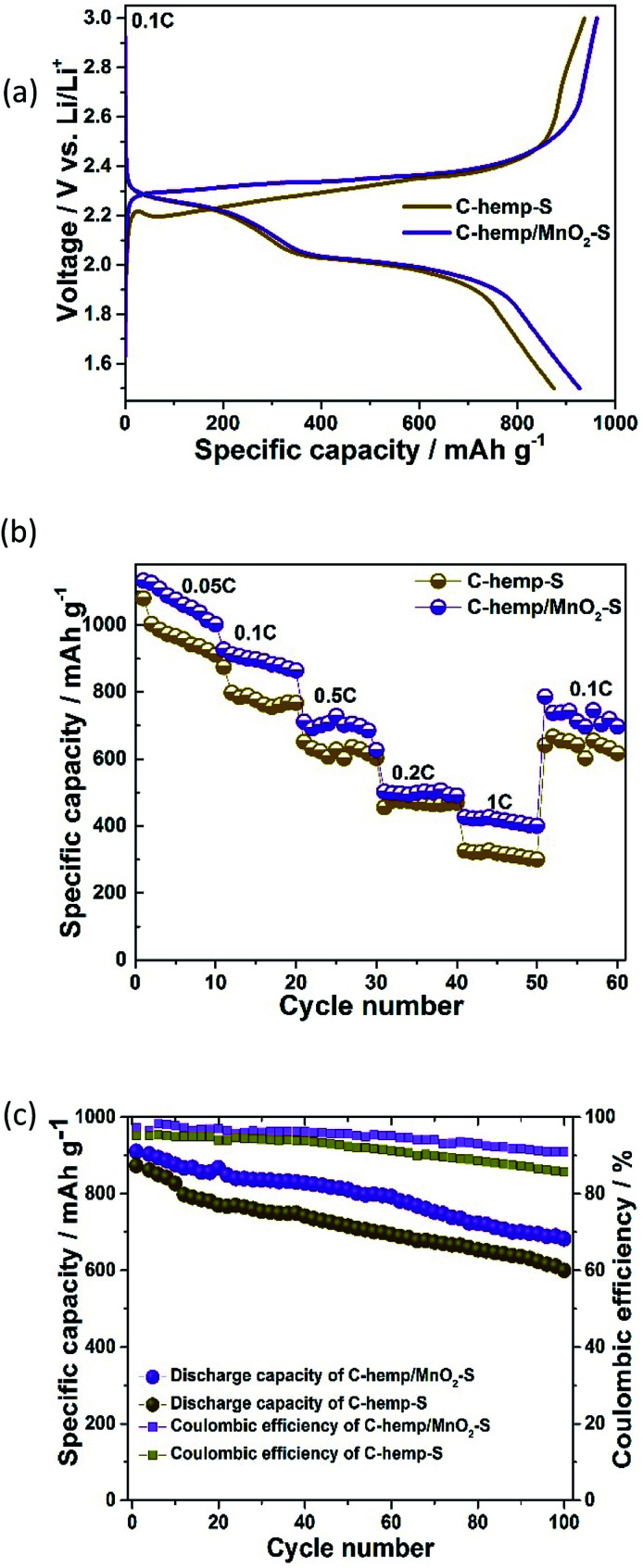
(a) Initial charge–discharge curves of C-hemp-S and C-hemp/MnO_2_–S at a current density of 0.1 C and (b) rate capability of C-hemp-S and C-hemp/MnO_2_–S at different current densities ranging from 0.05 C to 1 C. (c) Cycle performance and columbic efficiency of the C-hemp-S and C-hemp/MnO_2_–S electrodes at 0.1 C.

Fig. 7a describes the initial charge–discharge curves of C-hemp-S and C-hemp/MnO_2_–S at a rate of 0.1 C. They both have characteristic plateaus at 2.3 V and 2.0 V, which is in accordance with the earlier reports. The upper discharge plateau at 2.3 V corresponds to the reduction of S_8_ to long-chain polysulfides (Li_2_S_*n*_, 4 ≤ *n* < 8), while that at 2.0 V corresponds to the subsequent reduction of long-chain polysulfides to Li_2_S_2_/Li_2_S. C-hemp-S and C-hemp/MnO_2_–S deliver an initial discharge capacity of 874 and 927 mA h g^−1^, respectively, at a rate of 0.1 C. The increased capacity of C-hemp/MnO_2_–S indicates the polysulfide binding ability of the MnO_2_ nanoparticles scattered on the surface of the C-hemp matrix.

Fig. 7b represents the rate performance of the C-hemp-S and C-hemp/MnO_2_–S electrodes at different current densities. It is found that the discharge capacity of C-hemp-S is 1079, 874, 651, 455 and 326 mA h g^−1^ at the rates of 0.05 C, 0.1 C, 0.2 C, 0.5 C and 1 C, whereas the C-hemp/MnO_2_–S electrode delivers a capacity of 1131, 926, 711, 502, and 426 mA h g^−1^ at the same rates, respectively. When switched back to a rate of 0.1 C, C-hemp-S and C-hemp/MnO_2_–S deliver a capacity of 611 and 700 mA h g^−1^, respectively. However, the obtained value in the present study is lower compared with the previously reported values. This may be due to various factors including the structure of MnO_2_ and the properties of the carbon materials. Hence, further attention is needed to improve the cell characteristics, such as increase the specific capacity and cycle stability. A comparison of the present data with the literature is provided in Table S1 (ESI Table 1†). The cycling performance of C-hemp/MnO_2_–S and C-hemp-S at 0.1 C is shown in Fig. 7c. A capacity retention of 74% (675 mA h g^−1^) and coulombic efficiency of 91% are obtained over 100 cycles for C-hemp/MnO_2_–S, whereas for C-hemp-S, a capacity retention of only 68% with a coulombic efficiency of 86% is obtained.

**Fig. 8:**
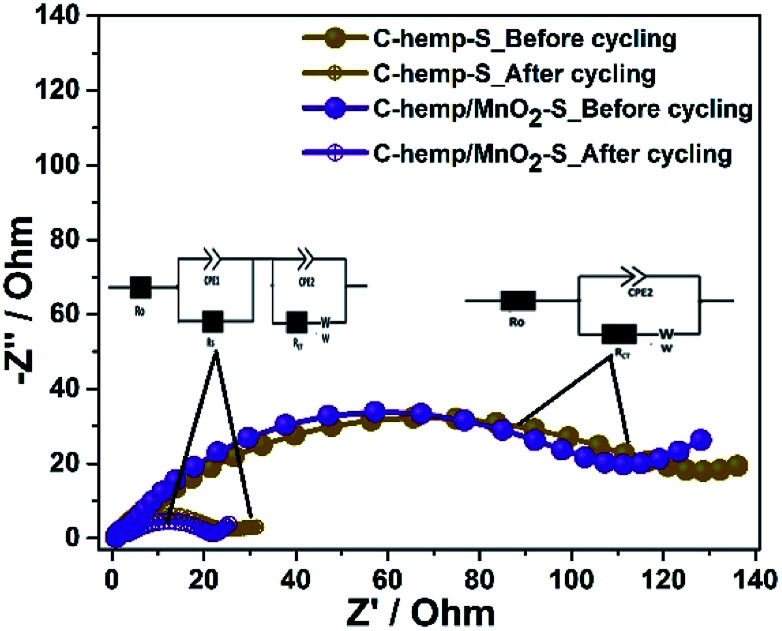
EIS spectra of C-hemp-S and C-hemp/MnO_2_–S cells before and after 100 cycles. Used equivalent circuits are shown in insets.

To retrieve further information regarding the electrochemical processes, electrochemical impedance spectroscopy was carried out. The Nyquist plots of both cells before the first discharge and after the 100th charge are given in Fig. 8(a and b). As seen in this figure, the impedance plots of the cells before cycling consist of a semi-circle in the high frequency region, which corresponds to charge-transfer resistance and interfacial impedance, while the inclined line at the low frequency zone corresponds to Warburg impedance (*W*). The real axis intercept in the high frequency region corresponds to the solution resistance. The Nyquist plots of the cells after cycling consist of an additional depressed semi-circle in the high frequency region, which corresponds to the resistance offered by the SEI layer. After cycling, the charge transfer resistance is found to decrease for both cells owing to the uniform distribution of sulfur as well as the availability of pores, which minimize the volume change. The reduction in charge-transfer resistance is greater for C-hemp/MnO_2_–S, which indicates more charge transfer between sulfur and the C-hemp/MnO_2_ material. The fitted impedance values are given in Table 1.

**Table 1:** Fitted impedance values of C-hemp-S and C-hemp/MnO_2_–S

	Impedance value	*R*_o_ (Ω)	*R*_s_ (Ω)	*R*_CT_ (Ω)
C-hemp-S	Before	1.4		107
	After	0.8	5.1	22
C-hemp/MnO_2_–S	Before	2.3		90
	After	0.4	5.8	11

**Fig. 9:**
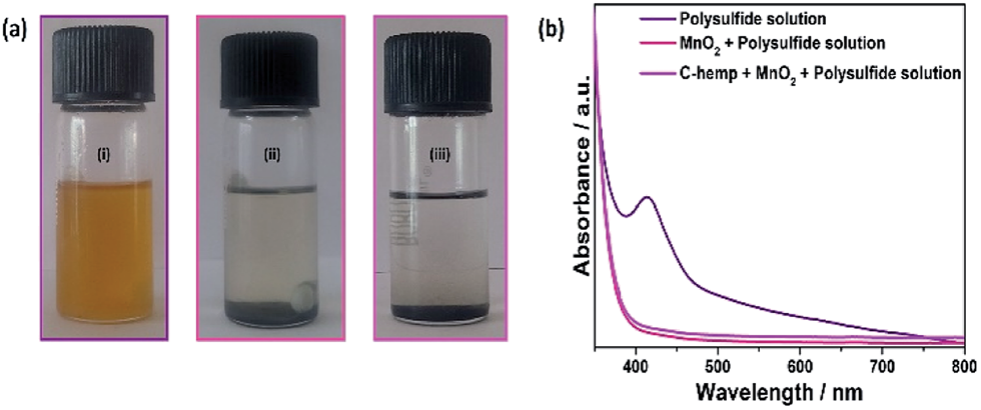
Photograph of (a) (i) lithium polysulfide solution, (ii) upon the addition of MnO_2_ and (iii) after the addition of C-hemp/MnO_2_. (b) Corresponding UV-visible spectra of the lithium polysulfide solution before and after the addition of MnO_2_ and C-hemp/MnO_2_.

To evaluate the interaction of MnO_2_ with polysulfides, around 60 mg MnO_2_ was added to 10 mL of 2 × 10^−2^ M Li_2_S_6_ in dimethoxyethane. The Li_2_S_6_ solution was initially yellow in colour (Fig. 9a(i)). Then, immediately upon contact with MnO_2_, it changed to light yellow and was completely colourless after 15 min, which indicates the strong adsorption of polysulfides (Fig. 9a(ii)). Also, the above solution became colourless upon the addition of C-hemp/MnO_2_ (Fig. 9a(iii)). Accordingly, the UV-visible absorption spectra of the above solutions were measured, which are presented in Fig. 9b. The broad peak at around 410 nm suggests the presence of higher order polysulfide anions. This distinct peak is absent for the solution after the addition of MnO_2_, which suggests the polysulfide adsorption ability of MnO_2_ nanoparticles.

## Conclusions

In the present study, a novel bio-source (hemp) was used for the synthesis of carbon, which was successfully employed as a sulphur host in the Li–S system. Further, amorphous MnO_2_ was synthesised *via* a simple solution process, which also showed the ability to accommodate sulphur in its structure. The combined effect of carbon and MnO_2_ leads to better polysulfide absorbing ability in the cathode matrix, which is the most promising finding in this study. This new C-hemp/MnO_2_–S composite significantly enhanced the specific capacity of the cell (926 mA h g^−1^ at 0.1 C) compared to the C-hemp/S composite (874 mA h g^−1^ at 0.1 C). The cell fabricated using C-hemp/MnO_2_–S showed better columbic efficiency (91%) and specific capacitance retention (74%) than the C-hemp-S composite even after 100 cycles at a rate of 0.1 C. Thus, the studied new C-hemp/MnO_2_–S composite is a potential cathode for the Li–S system. However, this study needs to be extended further for improving its cycle life by optimising the composite content.

## Conflicts of interest

There are no conflicts to declare.

## Supplementary Material

RA-008-C8RA03793K-s001
