# Supplementary material for: Low cost bio-derived carbon-sprinkled manganese dioxide as an efficient sulfur host for lithium–sulfur batteries
Source: RSC Adv. 2018 Jul 4;8(43):24261–7. doi: 10.1039/c8ra03793k (PMC9082114; doi:10.1039/c8ra03793k)
Supplement: RA-008-C8RA03793K-s001 [file RA-008-C8RA03793K-s001.pdf]

Electronic Supplementary Material (ESI) for RSC Advances.
This journal is © The Royal Society of Chemistry 2018

Supplementary Information

Low Cost Bio Derived Carbon Sprinkled Manganese Dioxide as an Efficient Sulfur Host for Lithium-Sulfur Battery

Aswathy Raghunandanan, Ulaganathan Mani, Ragupathy Pitchai*

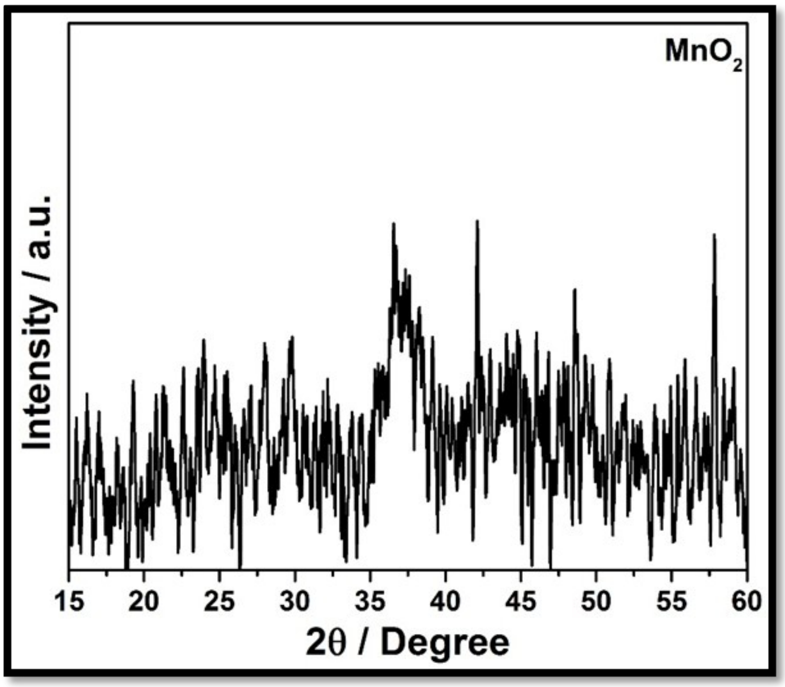

Fig. S1: XRD pattern of MnO_2

Table S1: Carbon-MnO_2 composite synthesized via various methods have been used as cathode in Li-S system

Sl. No.	Material	Specific capacity ($mAh\ g^{-1}$)	Cycle performance	Coulombic efficiency (%)	Reference
1	CNF-MnO_2-Sulfur	995 at 0.2C	600 $mAh\ g^{-1}$ after 400 cycles at 1C	99.66	1
2	Carbon-MnO_2 hollow nanofibers-Sulfur	1248 at 0.1C	0.050% capacity decay rate per cycle over 1000 cycles at 2.0 C	98	2

3	Core-shell γ-MnO_2-Sulfur	1350 at 0.1C	Fade rate of 0.07% per cycle over 600 cycles at 1 C	95	3
4	Dual Core–Shell-Structured S@C@MnO_2	1345 at 0.1C	Capacity decay rate of 0.052% per cycle after 1000 cycles at 3.0 C	98	4
5	3D porous reduced graphene oxide/ultrathin MnO_2 nanosheets-S aerogel composite	1360 at 0.1C	Capacity decay of 0.092% over 200 cycles at 0.2C	99	5
6	Sulfur–MnO_2@graphene	1416 at 0.1C	74% retention after 100 cycles at 0.2C		6
7	MnO_2@hollow carbon boxes-	1042 at 1 A g^{-1}	Stable over 500 cycles	100	7
8	GF/MWMCNT/MnO_2	1270 at 0.1C	824 mAh g^{-1} was retained after 160 cycles	99	8
9	Sulfur/manganese dioxide nanosheet		Capacity decay of 0.036%/cycle over 2,000 cycles	98	9
10	C-hemp/MnO_2-Sulfur	926 at 0.1C	74% retention over 100 cycles at 0.1 C	91	Present study

References

1 T. An, D. Deng, M. Lei, Q.-H. Wu, Z. Tian, M. Zheng and Q. Dong, *J. Mater. Chem. A*, 2016, **4**, 12858–12864.

2 L. Ni, G. Zhao, Y. Wang, Z. Wu, W. Wang, Y. Liao, G. Yang and G. Diao, *Chem. - An Asian J.*, 2017, **12**, 3128–3134.

3 L. Ni, Z. Wu, G. Zhao, C. Sun and C. Zhou, *Small*, 2017, 1–11.

4 and G. D. Lubin Ni, Gangjin Zhao, Guang Yang, Guosheng Niu, Ming Chen, *ACS Appl. Mater. Interfaces*, 2017, **9**, 34793–34803.

5 X. Zhao, H. Wang, G. Zhai and G. Wang, *Chem. - A Eur. J.*, 2017, **23**, 7037–7045.

6 G. Yuan, H. Jin, Y. Jin and L. Wu, *J Solid State Electrochem*.

7 S. Rehman, T. Tang, Z. Ali, X. Huang and Y. Hou, *Small*, 2017, 1700087.

8 P. Ragupathy, S. A. Ahad, P. R. Kumar, H.-W. Lee and D. K. Kim, *Adv. Sustain. Syst.*, 2017, **1**, 1700083.

9 X. Liang, C. Hart, Q. Pang, A. Garsuch, T. Weiss and L. F. Nazar, *Nat. Commun.*, 2015, **6**, 5682.
